# Supplementary material for: Bayesian refinement of protein structures and ensembles against SAXS data using molecular dynamics
Source: PLoS Comput Biol. 2017 Oct 18;13(10):e1005800. doi: 10.1371/journal.pcbi.1005800 (PMC5662244; doi:10.1371/journal.pcbi.1005800)
Supplement: S1 Archive — (BZ2) [file pcbi.1005800.s011.tar.bz2 › gromacs_4.62_bayesian_saxs_refi/share/html/online/gro.html]

gro file format


## gro file format

|  |  |
| --- | --- |
| Main Table of Contents | **VERSION 4.6** |
| GROMACS homepage | **Sat 19 Jan 2013** |

---

Files with the gro file extension contain a molecular structure in
Gromos87 format. gro files can be used as trajectory by simply
concatenating files. An attempt will be made to read a time value from
the title string in each frame, which should be preceded by
't=', as in the sample below.

A sample piece is included below:

```
MD of 2 waters, t= 0.0
    6
    1WATER  OW1    1   0.126   1.624   1.679  0.1227 -0.0580  0.0434
    1WATER  HW2    2   0.190   1.661   1.747  0.8085  0.3191 -0.7791
    1WATER  HW3    3   0.177   1.568   1.613 -0.9045 -2.6469  1.3180
    2WATER  OW1    4   1.275   0.053   0.622  0.2519  0.3140 -0.1734
    2WATER  HW2    5   1.337   0.002   0.680 -1.0641 -1.1349  0.0257
    2WATER  HW3    6   1.326   0.120   0.568  1.9427 -0.8216 -0.0244
   1.82060   1.82060   1.82060
```

Lines contain the following information (top to bottom):

- title string (free format string, optional time in ps after 't=')- number of atoms (free format integer)- one line for each atom (fixed format, see below)- box vectors (free format, space separated reals), values: v1(x) v2(y) v3(z) v1(y) v1(z) v2(x) v2(z) v3(x) v3(y),
        the last 6 values may be omitted (they will be set to zero).
        Gromacs only supports boxes with v1(y)=v1(z)=v2(z)=0.

This format is fixed, ie. all columns are in a fixed
position. Optionally (for now only yet with trjconv) you can write gro
files with any number of decimal places, the format will then be
n+5 positions with n decimal places (n+1
for velocities) in stead of 8 with 3 (with
4 for velocities). Upon reading, the precision will be
inferred from the distance between the decimal points (which will be
n+5). Columns contain the following information (from left to
right):

- residue number (5 positions, integer)- residue name (5 characters)- atom name (5 characters)- atom number (5 positions, integer)- position (in nm, x y z in 3 columns, each 8 positions with 3
          decimal places)- velocity (in nm/ps (or km/s), x y z in 3 columns, each 8 positions with 4
            decimal places)

Note that separate molecules or ions (e.g. water or Cl-) are regarded
as residues. If you want to write such a file in your own program
without using the GROMACS libraries you can use the following formats:

C format: "%5d%-5s%5s%5d%8.3f%8.3f%8.3f%8.4f%8.4f%8.4f" Fortran format: (i5,2a5,i5,3f8.3,3f8.4) Pascal format: This is left as an exercise for the user

Note that this is the format for writing, as in the above example
fields may be written without spaces, and therefore can not be read
with the same format statement in C.

---

http://www.gromacs.org
